# Quantitative Effects of Repeated Muscle Vibrations on Gait Pattern in a 5-Year-Old Child with Cerebral Palsy

**DOI:** 10.1155/2011/359126

**Published:** 2011-04-05

**Authors:** Filippo Camerota, Manuela Galli, Claudia Celletti, Sara Vimercati, Veronica Cimolin, Nunzio Tenore, Guido M. Filippi, Giorgio Albertini

**Affiliations:** ^1^Physical Medicine and Rehabilitation Division, Umberto I Hospital, Sapienza University of Rome, 00185 Rome, Italy; ^2^Dipartimento di Bioingegneria, Politecnico di Milano, P.za Leonardo da Vinci 32, 20133 Milano, Italy; ^3^IRCCS San Raffaele Pisana, Tosinvest Sanità, Via della Pisana 235, 00163 Roma, Italy; ^4^Institute of Human Physiology, Catholic University of Rome, 00168 Rome, Italy

## Abstract

*Objective*. To investigate quantitatively and objectively the effects of repeated muscle vibration (rMV) of triceps surae on the gait pattern in a 5-year-old patient with Cerebral Palsy with equinus foot deformity due to calf spasticity. *Methods*. The patient was assessed before and one month after the rMV treatment using Gait Analysis. *Results*. rMV had positive effects on the patient's gait pattern, as for spatio-temporal parameters (the stance duration and the step length increased their values after the treatment) and kinematics. The pelvic tilt reduced its anteversion and the hip reduced the high flexion evidenced at baseline; the knee and the ankle gained a more physiological pattern bilaterally. The Gillette Gait Index showed a significant reduction of its value bilaterally, representing a global improvement of the child's gait pattern. *Conclusions*. The rMV technique seems to be an effective option for the gait pattern improvement in CP, which can be used also in very young patient. Significant improvements were displayed in terms of kinematics at all lower limb joints, not only at the joint directly involved by the treatment (i.e., ankle and knee joints) but also at proximal joints (i.e., pelvis and hip joint).

## 1. Introduction

Cerebral palsy (CP) is the most common cause of physical disability in children, with a reported incidence of 2.0 ± 2.5 per 1000 live births. Children with CP may present a variety of motor problems. Some are directly related to the lesion in the central nervous system, influencing muscle tone, balance, strength, and selectivity (primary problems), whereas static muscle contractures and bony deformities (secondary problems) develop slowly over time in response to the primary problems and to growth [1]. In particular diplegic form of cerebral palsy, characterized by spastic, bilateral, neurological signs in four limbs, presents major lower limb impairment with gait abnormalities [2]. Therapeutic management of children with CP is the focus of considerable clinical resources in many countries, so that the evaluation of the efficacy for new and established treatments is imperative. The aims of management differ due to the severity of the involvement, the distribution of the motor problems, the maturation, and the needs for participation in society. The most commonly employed interventions are physiotherapy (including stretching, strengthening, and motor training), use of orthoses, serial casting, electrical stimulation, intramuscular injections of botulinum toxin type A (BTX-A) or phenol, and orthopaedic surgery.

Recently, a study based on Transcranial Magnetic Stimulation (TMS) demonstrated that a repeated muscle vibration (rMV) intervention can modify primary motor cortex (M1) plasticity, inducing an increase in short intracortical inhibition (SICI) of the vibrated muscle and an inverse pattern in its antagonist [3, 4]. Since an increase of SICI parallels motor functional recovery after stroke [5], the study was extended to evaluate motor cortical reorganization and clinical evolution after rMV in stroke patients [6, 7]. Marconi et al. [6] showed, by TMS, that rMV, combined with physiotherapy, reduced the resting motor threshold of M1 areas and increased SICI, while an SICI decrease was reported in the extensor muscles. These changes showed significant correlations with parallel reduction in muscle tonus and increase in motor function. rMV effects persisted, in all the tested patients, without any decrease throughout all the followup (2 weeks) after the end of rMV. Paoloni et al. [7] demonstrated that segmental MV integrated with physical therapy improved gait strategy in chronic stroke patients with foot drop, maybe as consequence of effective brain reorganization. Mechanisms underlying such effects are discussed in previous studies [5–10].

Since rMV technique resulted to be safe and comfortable in adults [5–11] and at the moment no studies were conducted on children, the aim of this paper report is to evidence quantitatively, throughout a longer period, possible rMV benefits on the gait pattern in a child with CP with equinus foot due to calf spasticity.

## 2. Methods

### 2.1. Patient Presentation

The patient was a male affected by tetraplegic CP, bilateral, neurological signs in four limbs with minimal functional involvement of upper limbs associated with no mental retardations. He was born at the 31st week of gestation; at birth 1 minute and 5 minute Apgar scores were 8. The birth weight was 1790 gr, and the length was 43 cm. He has respiratory distress for which he was kept in an incubator for 26 days after birth. The MRI showed a periventricular leukomalacia. 

At the moment of the intervention he was 5 years old (weight: 21 kg, height: 102 cm). He was able to walk on toes with a pattern of increasing hip and knee flexion for the spasticity at the adductor muscles, flexor, and triceps surae; no contractures were present on the lower limbs. He was level II of the Gross Motor Function Classification System: he walks without devices and is limited in walking outdoors and in the community, with no ability to perform gross motor skills. 

No antispasticity drugs or restraint therapy had been done in the previous six months, and he did not undergo surgical treatment on the lower limbs. He did physiotherapy five times a week. 

Throughout all the experiment duration, no antispasticity drugs were administered.

The study was approved by the Ethics Research Committee of the Institute. 

His parents gave their informed consent to the child's participation in the study.

The gait pattern of the subject was compared to an aged matched normality band composed of 20 healthy subjects (Control Group: CG; age: 4.3–8.2 years; weight: 31.7 ± 8.4 Kg; height: 132.2 ± 8.5 cm). Selection criteria for nondisabled subjects included no prior history of cardiovascular, neurological, or musculoskeletal disorders. They exhibited normal motor and intellectual development.

### 2.2. Experimental Setup

The patient underwent the rMV treatment and was examined with clinical assessment and with gait analysis (GA) before (PRE session) and one month after the treatment (POST session).

**Figure 1:**
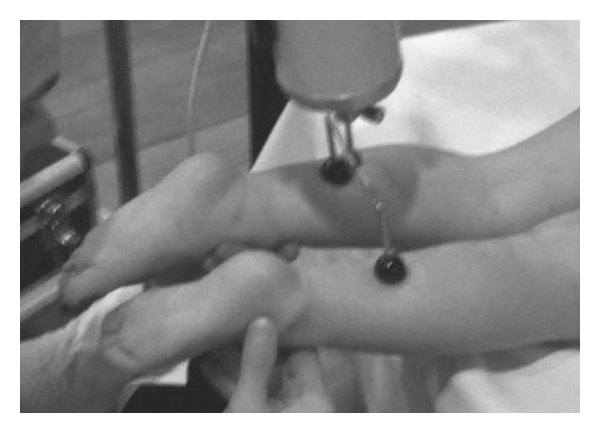
Picture representing the child during the treatment and the site of intervention.

GA was conducted using a 12-camera optoelectronic system (ELITE2002, BTS S.p.A., Milan, Italy) for the evaluation of movement kinematics and a synchronic video system (BTS S.p.A., Milan, Italy). After collection of the anthropometric measures (height, weight, tibial length, distance between the femoral condyles or diameter of the knee, distance between the malleoli or diameter of the ankle, and distance between the anterior iliac spines), passive markers were placed at special points of reference, directly on the subject's skin. The passive markers were positioned as described by Davis et al. [12], to acquire the movement of lower limbs and trunk, and in particular at C7, sacrum and bilaterally at the ASIS, greater trochanter, femoral epicondyle, femoral wand, tibial head, tibial wand, lateral malleolus, lateral aspect of the foot at the fifth metatarsal head, and at the heel (only for static offset measurement). After marker location the patient was asked to walk barefooted at his self-selected and convenient speed along a 10-metre walkway. At least five trials were recorded during each session in order to ensure consistency in the assessment of trials. Using a low amplitude (0.3–0.5 mm peak to peak), sinusoidal vibrations were administered at a fixed frequency of 100 Hz with a commercial mechanical device (Cro System, NEMOCO srl, Italy) over the triceps surae muscles (TS) in the prone position (Figure 1). This device has been previously described [5]. 

Vibration intervention was applied over 3 consecutive days, one session a day consisting of 3 × 10 min applications, separated by 1-minute interval. During the rMV the child was required to make a voluntary isometric contraction of vibrated muscle, against the hand of the assessor, to obtain about 30–50% of his plantarflexion. The clinical assessment was performed by the measurement of the dynamic ankle range of motion and passive range of motion in the supine position with the knee flexed to 90° and fully extended as assessed by the modified Tardieu Scale (MTS) [13]. The angle of full passive range of motion was designated R1. The ankle was dorsiflexed as fast as possible, and the catch angle at which the stretch reflex started was designated R2.

For this measurement, the child laid supine with the knee in full extension, and the subtalar joint stabilized. The ankle joint was flexed as fast as possible, and the first catch angle was measured by a manual goniometry. The degrees of dorsiflexion from the joint's neutral position were recorded as a positive number and the degrees of plantarflexion as a negative number. The results of the clinical assessment are displayed in Table 1.

### 2.3. Data Analysis

Some parameters were identified and calculated from kinematic data: spatiotemporal parameters and joint-angles values at specific instants of the gait cycle and range of motions (ROMs), calculated as difference between maximum and minimum values of a specific angle plot. In addition, in order to quantify globally the gait pattern of the subject, the Gillette Gait Index (GGI) or Normalcy Index (NI) [14, 15] was computed from GA data for each session.

### 2.4. Statistical Analysis

All the previously defined parameters were computed for all trials of the patient, and then the mean values and standard deviations of all indexes were calculated for the patient in each session and for the CG. Data of the first (PRE) and the second (POST) session were compared with the Wilcoxon tests, in order to detect significant changes in patient's gait pattern. The patient's and the controls' data were compared with Mann-Whitney *U* tests. Null hypotheses were rejected when probabilities were below 0.05.

## 3. Results

### 3.1. Clinical Evaluation (Table 1)

In PRE session the child was characterised by low ROM values of ankle passive dorsi-plantarflexion and of Tardieu test; the right side displayed less physiological values than the left one. One month later, in POST session, some improvements appeared mainly at the right side which gained higher joint excursion and more symmetric condition.

### 3.2. Gait Analysis (Table 2)

As concerns spatiotemporal parameters, before intervention the child walked with a lower velocity of progression, longer stance time, in particular on the right side, and reduced anterior step length, if compared to normal values: this condition was likely due to the search for better stability and equilibrium during gait. In the POST session these parameters, although far from normality, improved in terms of duration of right % stance, reducing the asymmetry present in the PRE session, and in terms of the bilateral anterior step length.

Statistically significant improvements were observed in the main joints kinematics. The pelvic tilts, which were characterised by an anterior position on the sagittal plane (Mean value index) and the presence of a double bump with a higher range of motion (ROM index), reduced the anteversion, getting closer to the control group values. However, the pelvic path maintained the double bump, and the range of motion (ROM index) remained unchanged.

As concerns the hip joint, before treatment it appeared flexed during the whole gait cycle, especially on the right side; it displayed an excessive right flexion at initial contact (IC index) and reduced bilateral extension ability in midstance (min in St); the maximum values in swing (Max Sw index) were higher than normal in both limbs. In POST session the IC and min St indices decreased at both limbs, and particularly on the right side. The maximum value in the swing phase (Max Sw index) improved bilaterally, even though the hyperflexion remained. 

The knee joint in PRE session was characterised by high flexion during the stance (IC and min St indices) and swing (Max Sw index) phases for the right limb, while the left knee was characterised by hyperextension during the stance phase and a reduced flexion ability in swing phase, showing a limited range of motion (ROM index). The intervention lead to bilateral positive effects: the right side reduced its high flexion in the stance phase (IC and min St indices), while the left one reached more physiological values at the initial contact and in swing phase (Max Sw index). On the left side the tendency to hyperextension persisted with an extension value (min St index) far from normality. The knee range of motion improved bilaterally. The effects of the treatment on ankle joints was a significant reduction of the plantarflexion on the left side during the whole gait cycle; on the right side, a better dorsiflexion ability in the swing phase (Max Sw index) was displayed. 

In terms of ankle joint, both feet were plantarflexed during the whole duration of the gait cycle (IC, Max St, min St, and Max Sw indices) with a more severe equinus foot at the left limb. The range of motion (ROM index) was higher than normality on the right side and limited on the left one.

**Figure 2:**
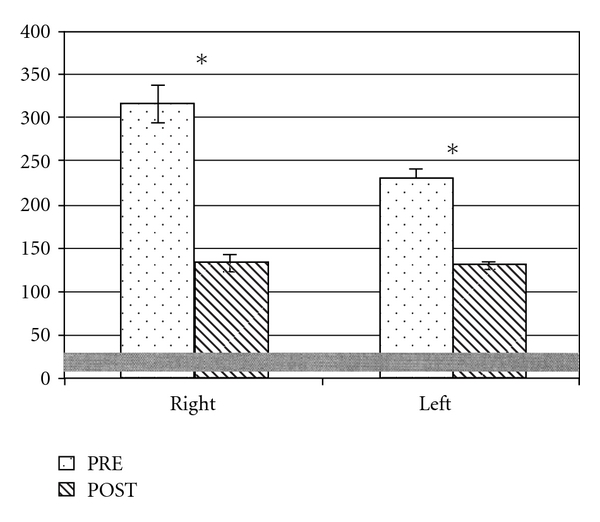
Comparison of GGI index (mean and standard deviation) for the subject (right and left side mean values with standard deviation) in the PRE and POST sessions; in grey the normative range is represented. *P < .05, PRE versus POST.

The GGI data (Figure 2) showed that in PRE session both sides were characterised by high values of this parameter, highlighting a severe global gait limitation (right: 315.4 ± 21.8; left: 230.4 ± 13.6; CG: 16.4 ± 9.8); one month after the treatment a significant reduction appeared bilaterally, although without reaching a physiological value (right: 133.8 ± 8.8; left: 131.9 ± 4.1; *P* < .05).

## 4. Discussion

The present research shows that a treatment based on short-term vibratory applications on calf muscle can induce a 1-month effect on the gait pattern of a very young child affected by CP. 

In our case study the evaluation of the rMV effectiveness on the gait patterns of a 5-year-old child with tetraplegia secondary to CP has been documented and quantified through the comparison of the child's gait before (PRE session) and one month after (POST session) the treatment. 

The examination of the PRE session data showed a severe alteration of the gait pattern bilaterally, both at proximal and distal joints. The child walked very slowly, with long stance duration and short step length. The pelvis was in an anterior position on the sagittal plane with high excursion during the gait cycle; the hip joint was shifted towards flexion, particularly for the right limb. The knee showed a strong asymmetric strategy: while the right side was characterised by a high flexion during the gait cycle, the left side displayed a hyperextended pattern in midstance. Both ankles were plantarflexed, and the left side showed a less physiological position than the other limb. The GGI mean values confirmed the global alteration of gait pattern for both sides. 

One month after the rMV therapy performed on the calf muscles (POST session) our data demonstrated a significant improvement in terms of spatiotemporal parameters and kinematics. Although far from normality, the duration of stance phase and step length improved, displaying a more symmetric gait pattern. The ankle improved mainly on the left side, approaching normality. At the proximal joints, the pelvic tilt and the hip pattern also improved, reasonably because of an increased ankle control. In particular the hip joint reduced its excessive flexion at the initial contact on the right side, and better extension ability in midstance was reached bilaterally. 

The strong asymmetry between the right and the left knees was reduced: while the left limb improved, maintaining the tendency to hyperextension in midstance, the right side reduced its excessive flexion during stance. As a result, the knee paths gained a quite physiological pattern.

Global improvements were confirmed by the GGI index, which reduced significantly its values bilaterally, showing a more physiological gait strategy. 

In conclusion, this paper demonstrated quantitatively and objectively the positive effects of the rMV application on gait pattern in a child with CP. In particular, GA showed that the effectiveness appeared on all lower limbs joints, not only at the joints directly involved in the treatment (i.e., ankle and knee joints) but also at the proximal joints (i.e., pelvis and hip joint). 

In particular the main improvement was noted at the left side which was characterised by the most severe equinus foot; as the treatment was applied with the same procedure and vibratory parameters, our results may demonstrate that the improvements appeared to be wider in presence of the highest degree of functional limitation.

The study has some limitations. Only one single case is reported, and this resulted in limited strength of the clinical and statistical findings. However, this paper represents the first attempt of rMV application in a child. Over the last few years, rMV procedure has received increasing scientific attention as a promising rehabilitation technique for adult chronic stroke [4, 7], although it has emerged only recently as a treatment approach for children. To our knowledge no quantitative evidences of its effects are present in children. At the moment the application of rMV on TS muscles for the reduction of equinus foot deformity in a patient with CP appeared to be effective, and its persistence may be longer than 2 weeks. rMV might be an important tool as a complementary/nonpharmacological therapy to promote prolonged neural plasticity and motor recovery also in children CP. In addition, not secondary, no muscle fatigue, pain or other side effects were observed during and after the treatment. 

Then only a muscles group, the TS, has been treated in this case report, being ankle dorsi-plantarflexion the most relevant deficit; obviously further researches may be conducted considering more muscle groups in patients with more complex functional limitation, where more than one muscle should be treated.

## Figures and Tables

**Table 1:** Clinical examinations of the ankle for the child in the PRE and POST sessions.

	PRE		POST	
	Right	Left	Right	Left
R1 with knee fully extended (°)	30	40	45	45
R2 with knee fully extended (°)	15	20	25	25
R1 with knee flexed at 90° (°)	15	25	25	30
R2 with knee flexed at 90° (°)	10	15	20	20

R1: full passive ankle range of motion; R2: ankle angle obtained after the dorsiflexion as fast as possible.

**Table 2:** Mean (standard deviation) values of the spatiotemporal and kinematic parameters for the child in the PRE and POST sessions and for the control group (CG).

	PRE		POST		CG
	Right	Left	Right	Left	
*Spatiotemporal parameters*					
Velocity (m/s)	0.2 (0.2)		0.3 (0.1)		1.2 (0.2)
Stance (%)	78.0 (1.2)	69.0 (2.0)	71.3 (2.1)*	72.3 (1.2)	59.5 (1.5)
Step length (mm)	181.0 (9.8)	189.0 (3.9)	303.3 (4.0)*	206.7 (3.5)*	546.2 (42.4)

*Pelvic tilt (°)*					
Mean value	28.1 (2.3)	29.7 (1.3)	17.2 (1.3)*	17.1 (2.3)*	8.8 (4.3)
ROM	12.8 (1.6)	11.8 (1.9)	12.8 (1.9)	11.0 (1.6)	1.6 (3.6)

*Hip flex-extension (°)*					
IC	48.8 (2.3)	30.4 (1.5)	39.3 (5.1)*	38.5 (5.2)	27.2 (7.5)
Min St	18.1 (2.5)	8.7 (1.9)	1.2 (2.7)*	−9.3 (2.7)*	−12.9 (7.6)
Max Sw	64.1 (1.4)	46.8 (2.3)	48.1 (0.7)*	61.4 (1.8)*	32.4 (7.1)

*Knee flex-extension (°)*					
IC	39.9 (2.4)	−5.1 (1.7)	29.4 (5.0)*	20.4 (6.1)*	6.7 (5.5)
Min St	13.8 (2.8)	−14.7 (2.4)	9.3 (4.8)*	−9.9 (2.1)	0.1 (3.8)
Max Sw	67.7 (3.6)	20.0 (2.6)	69.2 (4.5)	51.3 (1.7)*	56.3 (6.3)
ROM	53.9 (3.5)	34.6 (2.7)	61.3 (3.7)*	60.9 (3.0)*	55.8 (4.7)

*Ankle dorsi-plantarflexion (°)*					
IC	−1.2 (2.6)	−28.1 (2.8)	−0.6 (2.0)	1.9 (3.7)*	1.8 (5.8)
Max St	3.4 (3.8)	−28.1 (2.5)	5.6 (6.0)	4.9 (3.7)*	12.2 (5.5)
Max Sw	−10.6 (2.9)	−10.0 (2.1)	4.8 (3.1)*	2.4 (3.9)*	5.8 (6.5)
Min St	−34.7 (1.8)	−37.8 (3.2)	−32.4 (1.9)	−20.8 (3.1)*	−12.8 (5.9)
ROM St	38.1 (4.1)	8.8 (2.9)	38.1 (0.7)	25.3 (2.6)*	23.4 (4.8)

*P < .05, PRE versus POST.

IC: initial Contact; Max: maximum value; min: minimum value; St: stance phase; Sw: swing phase; ROM: range of motion.

## References

[1] Gage JR (2004). *The Treatment of Gait Problems in Cerebral Palsy*.

[2] Colver AF, Sethumadhavan T (2003). The term diplegia should be abandoned. *Archives of Disease in Childhood*.

[3] Marconi B, Filippi GM, Koch G (2008). Long-term effects on motor cortical excitability induced by repeated muscle vibration during contraction in healthy subjects. *Journal of the Neurological Sciences*.

[4] Brunetti O, Filippi GM, Lorenzini M (2006). Improvement of posture stability by vibratory stimulation following anterior cruciate ligament reconstruction. *Knee Surgery, Sports Traumatology, Arthroscopy*.

[5] Bütefisch CM, Netz J, Weßling M, Seitz RJ, Hömberg V (2003). Remote changes in cortical excitability after stroke. *Brain*.

[6] Marconi B, Filippi GM, Koch G (2011). Long-term effects on cortical excitability and motor recovery induced by repeated muscle vibration in chronic stroke patients. *Neurorehabilitation and Neural Repair*.

[7] Paoloni M, Mangone M, Scettri P, Procaccianti R, Cometa A, Santilli V (2010). Segmental muscle vibration improves walking in chronic stroke patients with foot drop: a randomized controlled trial. *Neurorehabilitation and Neural Repair*.

[8] Fattorini L, Ferraresi A, Rodio A, Azzena GB, Filippi GM (2006). Motor performance changes induced by muscle vibration. *European Journal of Applied Physiology*.

[9] Filippi GM, Brunetti O, Botti FM (2009). Improvement of stance control and muscle performance induced by focal muscle vibration in young-elderly women: a randomized controlled trial. *Archives of Physical Medicine and Rehabilitation*.

[10] Noma T, Matsumoto S, Etoh S, Shimodozono M, kawahira K (2009). Anti-spastic effects of the direct application of vibratory stimuli to the spastic muscles of hemiplegic limbs in post-stroke patients. *Brain Injury*.

[11] Christova M, Rafolt D, Mayr W, Wilfling B, Gallasch E (2010). Vibration stimulation during non-fatiguing tonic contraction induces outlasting neuroplastic effects. *Journal of Electromyography and Kinesiology*.

[12] Davis RB, Ounpuu S, Tyburski D, Gage JR (1991). A gait analysis data collection and reduction technique. *Human Movement Science*.

[13] Tardieu G, Shentoub S, Delarue R (1954). A la recherche d’une technique de mesure de la spasticité. *Revue neurologique*.

[14] Schutte LM, Narayanan U, Stout JL, Selber P, Gage JR, Schwartz MH (2000). An index for quantifying deviations from normal gait. *Gait and Posture*.

[15] Romei M, Galli M, Motta F, Schwartz M, Crivellini M (2004). Use of the normalcy index for the evaluation of gait pathology. *Gait and Posture*.

